# Anticoagulation After Ischemic Stroke or Transient Ischemic Attack (TIA) in the Time of Direct Oral Anticoagulation (DOAC) and Thrombectomy

**DOI:** 10.7759/cureus.17392

**Published:** 2021-08-23

**Authors:** Claudia Martin Diaz, Eduardo A Guizan Corrales, Starlie C Belnap, Jonathan Kline, Radhan Gopalani, Sylvia Marrero, Felipe De Los Rios La Rosa

**Affiliations:** 1 Pharmacy, Baptist Hospital of Miami, Miami, USA; 2 Miami Neuroscience Institute, Baptist Health South Florida, Miami, USA

**Keywords:** therapeutic anticoagulation, acute ischemic stroke, atrial fibrillation management, endovascular thrombectomy, thrombolytic therapy, direct oral anticoagulant therapy

## Abstract

Objective

To assess anticoagulation (AC) timing and appropriateness in patients with acute ischemic stroke (AIS) or transient ischemic attack (TIA) due to atrial fibrillation (AF) in a predominantly Hispanic community hospital in the era of direct oral AC (DOAC) and endovascular thrombectomy (EVT).

Methods

Adult patients presenting with known or new-onset AF and primary diagnosis of AIS/TIA admitted to Baptist Hospital of Miami between January 2018 and January 2019 were included. AC appropriateness was determined on medical history and concordance with American Heart Association AHA/American Stroke Association (ASA) AC guidelines. Median time from AIS/TIA diagnosis to AC initiation was the primary endpoint. AC guideline concordance on admission and at discharge, discordant justification, and AC selection were secondary endpoints.

Results

The sample included 120 patients. AC initiation was five days (interquartile range (IQR) 2-9) following AIS/TIA. Patients’ receiving intravenous (IV) alteplase experienced a 1.4-day delay in AC initiation (x̅=5.44, SE=1.05, p<.05). There was no significant delay for those receiving EVT. A symptomatic hemorrhagic transformation occurred in 3% (n=3) of patients; only one patient was initiated on AC prior to the event. No recurrent AIS/TIAs occurred prior to discharge. Guideline-based AC concordance increased by 14% to 96% from admission to discharge. Apixaban (78%, n=52) was the most prescribed anticoagulant during hospitalization.

Discussion

This study suggests that early AC initiation for patients with AF and AIS/TIA with or without IV alteplase and/or EVT is a safe and effective stroke prevention intervention. Further, it identified a need for improved concordance with guideline-based AC within the clinic setting.

## Introduction

Atrial fibrillation (AF), the most common cardiac arrhythmia in clinical practice, affects more than 2.3 million adults in the United States [1]. The principal adverse consequence of AF is acute ischemic stroke (AIS), and stroke is a leading cause of disability and morbidity [2]. Anticoagulation (AC) with vitamin K antagonists or direct oral anticoagulants (DOACs) reduces stroke risk up to 75% in patients with AF [3]. However, prescribers may be prone to initiating a sub-therapeutic or supra-therapeutic dose, increasing the risk for thromboembolic stroke or bleeding, respectively [4-5]. Known racial disparities in the use of anticoagulants further increase this risk for people of color [6-7].

Another challenge is optimizing AC timing following a stroke. Within the first 14 days following a stroke, patients are susceptible to recurrent ischemic stroke (RIS) and hemorrhagic transformation (HT) [3,8]. Published literature on RIS and HT risk after early AC initiation demonstrated mixed results. HT risk factors include the use of thrombolytics and/or AC, infarct size, endovascular thrombectomy (EVT), or bleeding diathesis [3]. United States atrial fibrillation guidelines recommend initiating AC regardless of formulation, intravenous (i.e. heparin infusion), or oral (i.e. warfarin, apixaban) within 14 days of stroke onset and that delaying AC beyond 14 days is reasonable for patients at high risk for HT [9]. In contrast, European guidelines recommend initiating AC according to stroke severity, characteristics, and size; for mild stroke (National Institutes of Health Stroke Scale [NIHSS] <8) after three days, for moderate strokes (NIHSS=8-16) after six days, moderate/severe stroke (NIHSS 8-16) after five to seven days, and severe stroke (NIHSS>16) after 12-14 days [10]. However, neither guideline considers which oral AC agent is initiated, or the difference in therapeutic effect onset between DOAC, a few hours, or warfarin, approximately five to seven days. To account for the therapeutic onset differences, both the US and European guidelines provide a bridging recommendation of warfarin with parenteral AC [9-10]. However, neither the bridging guidelines nor the initiation guidelines provide considerations for whether the patient underwent treatment with thrombolytics and/or EVT.

The present study evaluates the timing of AC initiation post-stroke in AF patients, AC dose appropriateness, the incidence of in-hospital RIS or HT, how treatment with IV alteplase or EVT influences these variables, and if these variables vary by race. Additionally, we evaluate prescribing patterns and adherence to clinical guidelines at a predominantly Hispanic community comprehensive stroke center.

## Materials and methods

A retrospective study was performed at a tertiary care certified comprehensive stroke center community hospital that serves a predominantly Hispanic population located in south Florida from January 2018 to January 2019. This study was reviewed by our institutional review board (IRB) committee and received approval under the process improvement guidelines. Patients admitted with signs and symptoms of AIS/TIA in our institution underwent brain imaging to exclude a hemorrhage and determine eligibility for IV alteplase. In addition, computerized tomography (CT) or magnetic resonance (MR) angiography (CTA or MRA, respectively) of the head and neck vessels was obtained when appropriate to confirm if large-vessel occlusions were present and to determine eligibility for EVT [11]. The National Institutes of Health Stroke Scale (NIHSS) evaluation tool was performed on admission and twice per day to assess the severity of stroke and stroke-related deficits [12].

Patients were selected if they were ≥ 18-years-old, admitted for an AIS/TIA, and had known AF or were diagnosed with AF during their hospital stay. Patients were excluded if they had intracranial hemorrhage (ICH) upon admission, if they were unable to tolerate brain imaging studies, or if they were pregnant. Time to AC initiation was measured in days from stroke admission to the time that the first dose was administered to the patient, as documented in the medication administration record. Anticoagulation administered within the first 24 hours was recorded as zero. For patients not started on AC during hospitalization, the recommended start date from the physician discharge summary was used to calculate AC initiation. Other items abstracted from the medical record included thrombolytic therapy with alteplase, EVT, or both, whether the patient experienced a recurrent stroke or symptomatic hemorrhagic transformation (SHT) while admitted, and if the patient was readmitted within 30 days secondary to hemorrhage or recurrent ischemic stroke. The presence of hemorrhagic transformation on brain CT or MRI imaging that led to death or a change in NIHSS score ≥4 was used to define SHT [13-14]. All data collection points are viewable in Appendix. Appropriateness of AC on admission and discharge was based on patients past medical history (e.g., recurrent falls with injuries and/or bleeding episodes, CHA2DS2-VASc (Congestive heart failure, Hypertension, Age, Diabetes, previous Stroke/transient ischemic attack - vascular disease) and HAS-BLED (HAS-BLED) scores, history of AF, age, renal function, weight, and central nervous system disease affecting the ability to take medications) and whether AC prior to presentation with AIS/TIA is warranted according to current guidelines [9-11].

Data points of interest were visually inspected and found to be non-linear. Therefore, all statistical evaluations used non-parametric methods. Admission data, index stroke features, and discharge features were evaluated by race using the Kruskal-Wallis non-parametric tests and the Pearson chi-square test. Significant findings were further evaluated using the Mann-Whitney U test and the pairwise Pearson chi-square for posthoc comparisons. Bonferroni correction was used for multiple comparisons. Median, interquartile range (IQR), and adjusted p-value are reported for all continuous variables while count, percentages, and p-value are reported for categorical variables. The primary objectives were analyzed using a generalized linear model (GLM) using a Poisson distribution with a log link. Admission variables and index stroke features were used to evaluate the timing of AC initiation. Factors with high collinearity were excluded from the model. For the analysis of the secondary objectives, a GLM using binomial distribution and logit link estimated which factors influenced the likelihood of appropriately following AC guidelines upon admission and upon discharge. When evaluating factors at admission, only admission features were included in the model, whereas at discharge, admission data and index stroke features were included in the model. Chi-square tests evaluated changes in AC guideline concordance between admission and discharge and in anticoagulant type. Mean, standard error, coefficient, and effect size were reported when appropriate. All testing was conducted at an alpha value of 0.05, with Bonferroni corrections set at 0.017 when applicable.

## Results

**Figure 1:**
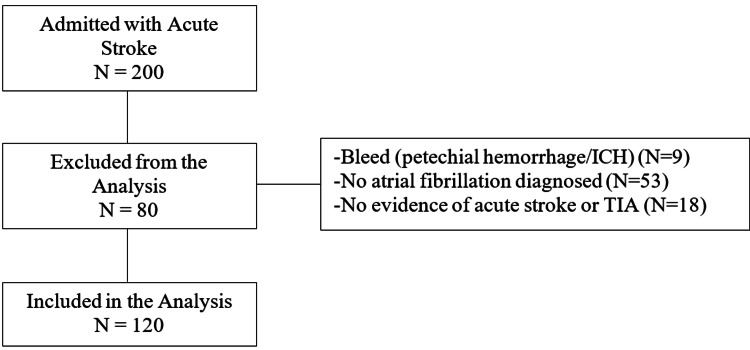
Subject Selection Description of the subject inclusion and exclusion criteria.

During the study time window, 200 patients were admitted with symptoms of an AIS/TIA. Eighty patients were excluded from the final analysis; 53 patients did not have AF, 18 patients had no documented evidence of AIS/TIA, and nine patients presented with a hemorrhagic stroke or petechial hemorrhage on admission and were deemed ineligible for AC (Figure 1). Patients meeting inclusion criteria totaled 120. Of these, 67 (55.8%) were initiated on AC after the AIS/TIA during their hospital stay, 29 (24.2%) had a documented plan for AC initiation after discharge; nine (7.5%) patients had no documented time period for AC initiation, and 15 (12.5%) were changed to comfort measures only (CMO), hospice care, expired during hospitalization, or refused treatment.

Patient clinical characteristics are listed in Table 1. The majority of patients were female (53%, n=63) with a median age of 83 (54-97). The majority of the sample was Hispanic with 53% (n=64) of patients listed as Hispanic, 36% (n=43) listed as non-Hispanic White (NHW), and 11% (n=13) were listed as non-Hispanic Black (NHB). Nearly 86% of patients had a history of hypertension (n=103), which significantly varied by race (χ2(2)=7.92, p<0.05). Hispanic patients were more likely to have a hypertension diagnosis prior to admission when compared to NHW patients (χ2(1)=7.97, p<0.01) but not when compared to NHB patients (χ2(1)=1.25, p=0.26). Approximately, 73% (n=88) of our study sample had a history of AF, but AC prescriptions were only noted for 52% (n=46) of patients prior to admission, which did significantly vary by race (χ2(2)=6.04, p<0.05). Hispanic patients with a history of AF were less likely to be on AC prior to admission compared to NHW patients (χ2(1)=5.64, p < 0.05) but not when compared to NHB patients (χ2(1)=1.36, p=0.24). The median time of AC initiation was five days (IQR 2-9); this did not significantly vary by race. In general, 94% (n=113) presented with an acute ischemic stroke, and 6% (n=7) were diagnosed with a TIA. The median length of stay was six days (IQR 4-9) and the most prescribed anticoagulant during hospitalization was apixaban (78%, n=52), with 65% (n=34) of orders placed by the hospitalist. Lastly, for patients not started on AC during hospitalization, apixaban (79%, n=23) was the most recommended AC to begin after discharge.

**Table 1:** Clinical characteristics between race groups NHW=non-Hispanic White; NHB=non-Hispanic Black AF=atrial fibrillation; HAS-BLED=hypertension, abnormal renal and liver function, stroke bleeding, labile, INR, elderly, drugs or alcohol; IQR=interquartile range; NIHSS=National Institutes of Health Stroke Scale; AC=anticoagulant; a=p<0.05 when compared to the Hispanic group; b=p<0.05 when compared to the NHW group

	Hispanic N=64	NHW N=43	NHB N=13	P-Value
Features at Admission				
Age, y, median (IQR)	82(74-89)	84(79-89)	82(79-86)	.70
Female sex, n (%)	33 (52)	24 (55.8)	6 (46.2)	.81
Body weight, kg, median (IQR)	78(64-86)	74(57-85)	79(51-89)	.63
Hypertension, n (%)	60(93.8)	32(74.4)^a^	11(84.6)	.02
Dyslipidemia, n (%)	25(39.1)	12(27.9)	3(23.1)	.35
Diabetes mellitus, n (%)	21(32.8)	8(18.6)	3(23.1)	.25
Coronary Artery Disease, n (%)	14(22)	13(30.2)	4(30.8)	.57
AF diagnosis before event, n (%)	49(76.6)	31(72.1)	8(61.5)	.52
Premorbid anticoagulation, n (%)	31(48.4)	11(25.6)^a^	4(30.8)	.05
Premorbid antiplatelet, n (%)	26(40.6)	14(32.6)	3(23.1)	.42
Renal Function				
Creatinine Clearance, mL/min; median (IQR)	47(36-66)	39(33-53)	25(17-43)^a,b^	.001
SCr mg/dL, median (IQR)	1(.82-1.14)	1.1(.92-1.4)^a^	1.6(1.15-4.5)^a,b^	.001
Features of Index stroke				
Ischemic stroke, n(%)	61(95.3)	39(90.7)	13(100)	.39
Stroke Location				
Posterior, n (%)	23(35)	12(27.9)	2(15.4)	.49
Anterior, n (%)	40(63)	26(60.5)	11(84.6)	.27
Initial NIHSS score, median (IQR)	6 (1-12)	5 (2-13)	8 (5-16)	.32
Intravenous thrombolysis, n (%)	14(22)	13(30.2)	2(15.4)	.45
Endovascular therapy, n (%)	16(25)	9(20.5)	3(23.1)	.89
CHA_2_DS_2_-VASc score, median (IQR)	4(3-5)	4(3-5)	5(4-6)^b^	.12
CHA_2_DS_2_-VASc score ≥5, n (%)	25(39)	17(39.5)	8(61.5)	.31
HAS-BLED score, median (IQR)	4(4-5)	4(3-4)	5(4-5)^a,b^	.007
Days from onset to AC start, d, median (IQR)	4 (2-10)	4.5 (1-7)	6.5 (2-15)	.33
Features at Discharge				
Discharge NIHSS score, median (IQR)	2.5(1-6)	1(0-5)	5(1-13)	.14
Length of Stay, d, median (IQR)	6 (4-10)	5 (3-9)	8 (7-12)^b^	.06
Discharge Status				
Home, n (%)	30(47)	21(48.8)	3(23.1)	.24
Acute Inpatient Rehab, n (%)	14(22)	6(14)	3(23.1)	.55
Long Term Care Facility, n (%)	13(20)	10(23.3)	5(38.5)	.37
Morality, n (%)	5(7.8)	2(4.7)	1(7.7)	.80
Anticoagulation at discharge, n (%)	37(57.8)	27(62.8)	6(46.2)	.72

Nine elderly patients (range from 74 to 94 years of age; average 85 years old) were not started on AC at discharge and did not have a documented recommended AC start date. Most were male (8/9) and Hispanic (6/9) and had an average admission NIHSS score of 10.2 and 6.3 at discharge. One patient received both thrombolytic and EVT and later experienced an asymptomatic HT; only one patient received thrombolytic alone. Most patients had hypertension (8/9), three patients had coronary artery disease, three had dyslipidemia, four had diabetes, and one patient had no documented comorbidities besides atrial fibrillation. Reasons documented for not initiating AC at discharge or having a planned start date ranged from extensive hematoma following a traumatic fracture of the hip, multiple arteriovenous malformations, asymptomatic HT, advanced dementia with recurrent falls, recurrent gastrointestinal bleed, family and patient preference, severely advanced Parkinson’s disease, and recent history of catheterization with three stents and transcatheter aortic valve replacement procedure (patient required double antiplatelet therapy).

Primary outcome

**Figure 2:**
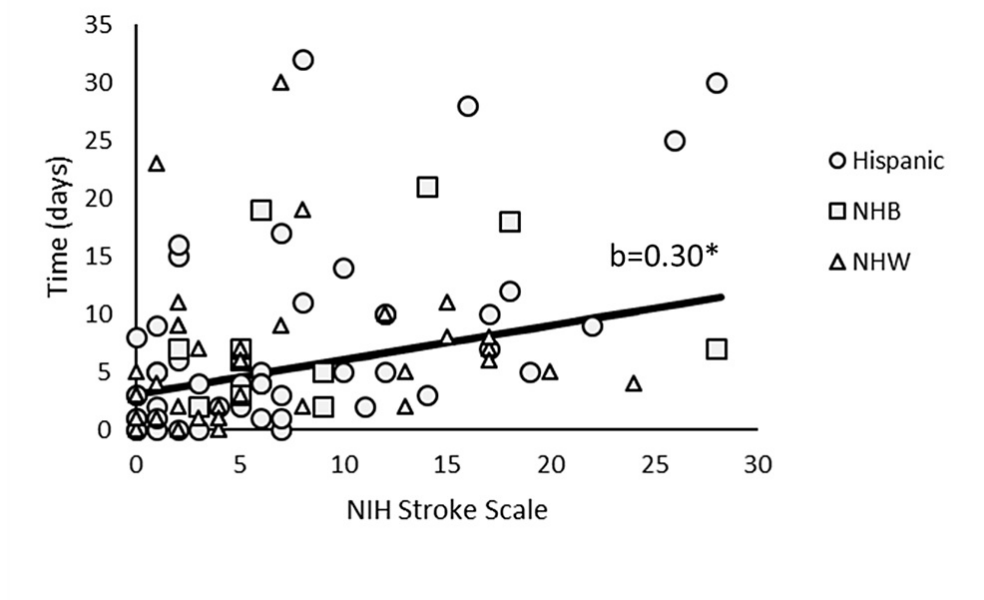
AC Initiation and NIHSS at Admission Illustrates the positive statistically significant relationship between AC initiation as measured in days and the NIHSS upon admission. Symbols represent a single patient. b=the regression coefficient. *p<0.05. AC: anticoagulation; NIHSS: National Institutes of Health Stroke Scale

The Poisson GLM model with admission data and index stroke features on AC initiation was significant (χ2(19)=228.15, p<0.001) compared to the base model. Age (χ2(1)=14.29, p < 0.001), sex (χ2(1)=17.20, p < 0.001), serum creatinine (SCr) (SCr mg/dL (χ2(1)=13.85, p<0.001), NIHSS (χ2(1)=16.70, p<0.001), HTN (χ2(1)=10.17, p<0.001), CAD (χ2(1)=9.83, p<0.01), and AF (χ2(1)=15.87, p<0.001) all showed independent statistically significant relationships with AC initiation. Age had a negative relationship with AC initiation (b=-.02, p<0.001); as age increased, the time to AC initiation decreased. On average, female patients (x̅=4.9, SE=0.97) were initiated earlier compared to male patients (x̅=7.56, SE=1.44). SCr (b=-.36, p<0.001) had a significant negative relationship with AC initiation; as SCr increased, the time to AC initiation decreased. NIHSS upon admission was positively associated with AC initiation (b=.30, p<0.001) as illustrated in Figure 2. For every one-unit increase in the NIHSS, there was approximately a 30% delay in AC initiation. Patients with a history of HTN (x̅=8.38, SE=0.96) and AF (x̅=8.62, SE=1.0) were started sooner on AC compared to those without a history of HTN (x̅=11.5, SE=1.72) and AF (x̅=10.93, SE=1.50); whereas patients with a history of CAD (x̅=11.49, SE=1.53) were likely to experience AC initiation delays compared to those with no CAD history (x̅=8.21, SE=1.03).

When considering the index stroke features, HAS-BLED (χ2(1)=15.33, p<0.001) and CHA2DS2-VASc (χ2(1)=4.88, p<.05) scores significantly contributed to AC initiation. For a one-unit increase in HAS-BLED score, there was a 24% delay in AC initiation (b=.24, p<0.001), and for a one-unit increase in CHA2DS2-VASc score, there was a 9% delay in AC initiation (b=.09, p<.05). Stroke type also influenced AC initiation (χ2(1)=5.58, p<.05). Patients who had AIS (x̅=9.16, SE=0.85) on average experienced a five-day delay in AC initiation when compared to patients who had TIA (x̅=1.04, SE=1.41). Similarly, the administration of IV alteplase influenced AC initiation (χ2(1)=4.45, p<0.05). Patients who received IV alteplase (x̅=6.80, SE=1.33) on average experienced a 1.4-day delay in AC initiation when compared to patients who did not receive IV alteplase (x̅=5.44, SE=1.05). Although patients who received EVT (x̅=6.4, SE=1.30) did experience, on average, a 0.7-day delay in AC initiation compared to patients who did not receive EVT (x̅=5.7, SE=1.08); this delay was not statistically significant (χ2(1)=0.77, p=0.38).

Secondary outcomes

Twenty-two patients (18%) had indications for AC but were not prescribed any agent on admission. All of these patients had a history of AF diagnosis and no previous history of bleeding, recurrent falls with injuries, or documented memory issues. Nineteen patients had indications for AC but were not prescribed an anticoagulant; three patients (2.5%) were on an AC dose not concordant with guidelines. Two were on the lower dose, apixaban 2.5 mg twice daily, but qualified for the higher dose, apixaban 5 mg twice daily. The remaining patient was on the higher dose of apixaban, however, qualified for the lower dosing regimen due to age and weight. Ninety-eight patients (82%) were on an appropriate AC dosing regimen upon admission.

**Figure 3:**
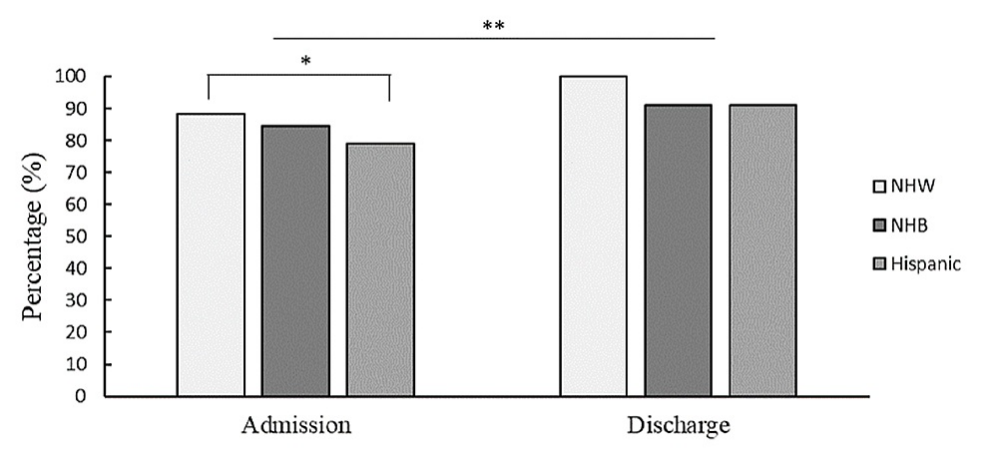
Percentage of Cases Following AC Guidelines Percentage of cases accurately following the AC guidelines upon admissions (left side) and upon discharge (right side) by race category. Non-Hispanic White (NHW) illustrated in white, Non-Hispanic Black (NHB) illustrated in dark gray, Hispanic illustrated in light gray. *p<.05 **p<.01

When considering admission criteria for AC appropriateness, the binomial GLM model revealed race (χ2(2)=4.89, p<0.05) and AF history (χ2(1)=4.61, p<0.05) significantly contributed to the model while HTN (χ2(1)=3.57, p=0.06) approached statistical significance. Hispanics patients were 12% more likely to be discordant with AC guidelines on admission when compared to NHW patients (b=-1.63, p<0.05) and, although not statistically significant, 6% more likely to be discordant when compared to NHB patients (b=-.58, p=.57) (see Figure 3). Patients with AF history were 20% more likely to be discordant with AC guidelines on admission compared to patients with new-onset AF (b=-2.32, p<.05). In contrast, patients with an HTN history were 12% more likely to be concordant with AC guidelines compared to those without an HTN history (b=1.53, p<.05).

At discharge, only four (3.9%) patients had AC indications but were not prescribed an anticoagulant. All four patients were Hispanic, had a history of HTN, experienced AIS, and did not have a history of CAD or DM. One patient was treated with thrombolytics and two patients received EVT. Three patients (2.5%) were under-dosed at discharge (apixaban 2.5 mg twice daily) mostly due to the perceived high risk of HT. The remaining patient was never initiated on AC but met the guidelines for AC initiation. Results revealed the majority of patients (n=99; 96.1%) were concordant with AC guidelines, reflecting a 14.1% statistically significant improvement in guideline adherence ((χ2(1)=7.33, p < 0.01) when compared to admission (see Figure 3). The binomial GLM model with discharge concordance revealed that race, HTN, and AF history no longer influenced AC guideline concordance at discharge, nor did stroke type (i.e. AIS & TIA), stroke location, or stroke treatment (i.e. thrombolytics & EVT).

Symptomatic hemorrhagic transformation occurred in 2.5% (n=3) of patients, and there was no recurrent stroke noted during admission. Patients that suffered an SHT were 74, 79, and 88-years-old with an admission NIHSS score of 0, 14, and 20, respectively. The 79-year-old patient was the only one that received IV alteplase and all three underwent EVT. Additionally, all three patients were on guideline-concordant AC on admission, however, only the 74-year-old patient had AC re-initiated prior to the SHT, approximately three days after admission. For this sample, 45% (n=45) were discharged home, 19% (n=23) were discharged to an acute inpatient rehabilitation facility, 24% (n=29) were discharged to a long-term care facility, 5% (n=6) were discharged on hospice, and 8% (n=8) expired. AC concordance upon admission or discharge was not significantly related to discharge destination or mortality.

## Discussion

This current study demonstrated a median time to AC initiation of five days post-stroke in a predominately Hispanic population, a period within the recommended guideline time frame of 4-14 days [11]. This approach was found to be safe with no recurrent ischemic events and rare SHT, despite 88% of anticoagulants prescribed during inpatient care being DOACs (52 patients on apixaban, four patients on rivaroxaban, three patients on dabigatran) and the remaining 12% on either warfarin, heparin continuous infusion, or therapeutic dose of enoxaparin. IV alteplase, EVT, or the combination of both delayed AC initiation by approximately one day and was not found to increase the risk of hemorrhagic complications. One factor that potentially contributed to earlier AC initiation was moderate median NIHSS scores with a presumed lower risk of hemorrhagic complications. Our findings are consistent with recent studies where early AC initiation was associated with lower rates of recurrent AIS, ICH, and death [3,8]. There were no in-hospital recurrent strokes observed in this study, which is significantly lower than previously reported rates of 8% in the first two weeks [9]. In contrast, the incidence of SHT was noted to be 3%, which is comparable to rates observed in the literature [8].

Despite the complexities of oral AC dosing, this study demonstrated that the majority of patients were appropriately dosed at hospital admission and discharge. However, as documented in other studies, anticoagulation continues to be under-prescribed in the outpatient setting [4-5]. In our analysis, 18% of patients with atrial fibrillation and a stroke or TIA admission were not appropriately prescribed anticoagulation in the outpatient setting. It is noteworthy that 23.4% of Hispanic patients with preadmission AF were not on an anticoagulant when according to the AC guidelines, they should have been. This was similar to NHB but higher than NHW. This finding suggests that racial disparities in regards to anticoagulation for the prevention of stroke continue to persist in the community [6-7]. Additional studies are required to confirm and clarify the reasons for this finding since racial differences in AC prescription were not evident at hospital discharge. Lastly, the population included in this study represents high-risk patients with AF who benefit from AC, with a median age of 83 years and a median CHA2DS2-VASc score of 4. Patients in this cohort experienced moderate strokes as reflected by a median admission NIHSS score of 10 on admission and 6 at discharge and those with HT were excluded. Therefore, our findings cannot be extrapolated to other stroke populations. Our analysis is retrospective and subject to selection bias, therefore, careful consideration should be given to early initiation of AC in patients with additional bleeding risks.

The retrospective, single-center study design limited the sample size. Data collection relied exclusively on existing documentation available in the electronic health record as well as extrapolation/assumption of AC initiation post-discharge. Furthermore, the short hospital length of stay (median of 6 days) reduced the chance to capture all recurrent stroke or bleeding events, which are at the highest risk within the first two weeks after an AIS. However, it would be expected that patients experiencing SHT or recurrent AIS would require hospital re-admission, which would have been captured by this analysis as long as readmission happened within our hospital system. This analysis does not include stroke size as a study variable. The NIHSS does correlate with stroke size; however, there is significant variability in the volume of infarcted brain tissue for a given NIHSS depending on the territory that is affected (eloquent vs. non-eloquent) and laterality (larger stroke volumes for a given NIHSS score on right-sided strokes) [15].

## Conclusions

This study suggests that early AC initiation with DOACs in patients with AF who experience a moderate AIS, as defined by the NIHSS, is a safe and effective secondary stroke prevention intervention and is not associated with a significantly increased risk of HT in a predominately Hispanic population. Although thrombolytic therapy slightly delayed AC initiation, we found no additional safety concerns. Even though anticoagulation dosing was appropriate in the vast majority of cases, there are still racial disparities and missed opportunities to start anticoagulation for stroke prevention in the outpatient setting.

## Appendices

Data points collected

·         Age

·         Weight

·         Race

·         Renal function (creatinine clearance calculated with the Cockcroft-Gault equation and serum creatinine)

·         History of antiplatelet use prior to admission

·         Type of anticoagulation ordered, dose, and frequency on admission

·         Specialty of physician ordering anticoagulation

·         History of atrial fibrillation or new diagnosis

·         NIHSS score on admission and discharge

·         Location of stroke per brain CT or MRI

·         Presence of HT (symptomatic vs asymptomatic)

·         Thrombolysis therapy, endovascular thrombectomy, or both, as well as any other surgery

·         HAS-BLED and CHA2DS2VASc scores

·         Length of stay

·         In-patient mortality

·         Discharge status (home, hospice, long-term care facility, skilled nursing facility, etc.)
